# Alternative Splicing in Cardiovascular Disease—A Survey of Recent Findings

**DOI:** 10.3390/genes12091457

**Published:** 2021-09-21

**Authors:** Ena Hasimbegovic, Victor Schweiger, Nina Kastner, Andreas Spannbauer, Denise Traxler, Dominika Lukovic, Mariann Gyöngyösi, Julia Mester-Tonczar

**Affiliations:** Division of Cardiology, Department of Internal Medcine II, Medical University of Vienna, A-1090 Vienna, Austria; ena.hasimbegovic@meduniwien.ac.at (E.H.); victor.schweiger@student.i-med.ac.at (V.S.); nina.kastner@meduniwien.ac.at (N.K.); andreas.spannbauer@meduniwien.ac.at (A.S.); denise.traxler@gmail.com (D.T.); dominika.lukovic@meduniwien.ac.at (D.L.); julia.mester-tonczar@meduniwien.ac.at (J.M.-T.)

**Keywords:** alternative splicing, atherosclerosis, dilated cardiomyopathy, myocardial infarction, circular RNAs, heart failure

## Abstract

Alternative splicing, a driver of posttranscriptional variance, differs from canonical splicing by arranging the introns and exons of an immature pre-mRNA transcript in a multitude of different ways. Although alternative splicing was discovered almost half a century ago, estimates of the proportion of genes that undergo alternative splicing have risen drastically over the last two decades. Deep sequencing methods and novel bioinformatic algorithms have led to new insights into the prevalence of spliced variants, tissue-specific splicing patterns and the significance of alternative splicing in development and disease. Thus far, the role of alternative splicing has been uncovered in areas ranging from heart development, the response to myocardial infarction to cardiac structural disease. Circular RNAs, a product of alternative back-splicing, were initially discovered in 1976, but landmark publications have only recently identified their regulatory role, tissue-specific expression, and transcriptomic abundance, spurring a renewed interest in the topic. The aim of this review is to provide a brief insight into some of the available findings on the role of alternative splicing in cardiovascular disease, with a focus on atherosclerosis, myocardial infarction, heart failure, dilated cardiomyopathy and circular RNAs in myocardial infarction.

## 1. Introduction

**Figure 1:**
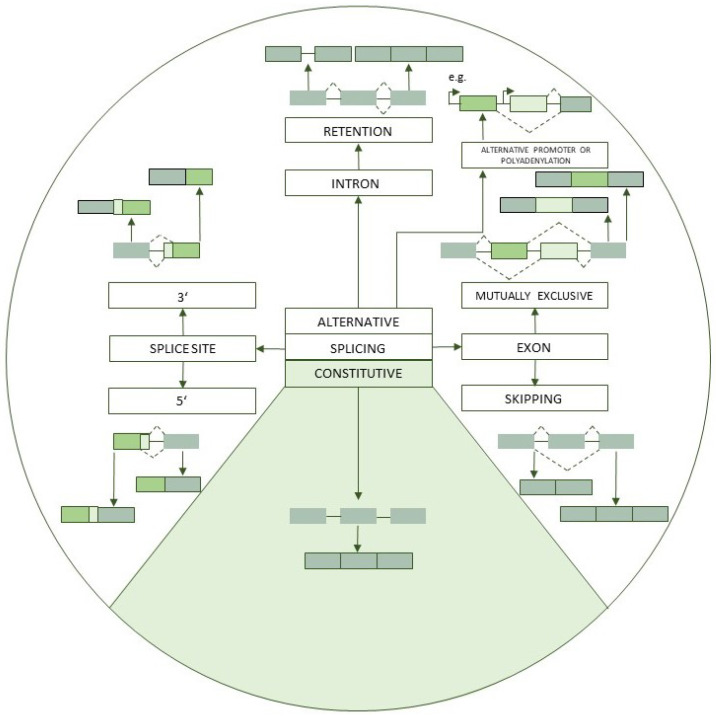
Alternative splicing mechanisms. The green rectangles represent the exons, the horizontal lines connecting the rectangles represent the introns. Arrows point to the products of the respective splicing event. The constitutive splicing mechanism is represented in the light green section of the diagram. The different shades of green in the rectangles are used to accentuate the segment affected by a certain alternative splicing mechanism.

Initially transcribed immature pre-mRNA transcripts are further altered through the splicing process. During splicing, intronic sequences are removed from the primary transcript and the exons are ligated to form the mature mRNA transcript [1]. Alternative splicing, the set of pathways that utilize alternative splice sites, mutually exclusive exons, exon inclusion/exclusion and the retention of intronic sequences, results in the formation of multiple protein isoforms from the same gene [1,2,3]. The mechanisms of alternative splicing are illustrated in Figure 1.

Modern high-throughput methods have delivered new insights regarding the frequency of alternative splicing events in humans, which have been found to occur in nearly all multiple-exon genes [4]. Alternative splicing events can play out in a tissue-specific manner and play a role in the development of tissues and organs [5,6]. This is also true for human heart development [7]. Alternative splicing has been recognized as a key source of transcriptome diversity and is thought to have played a significant role in the evolution of phenotypic and interspecies variability [8]. Alternative splicing has been implicated in the pathophysiology of various diseases, from cancer to neurological disorders [9,10]. This review aims to present some of the recent discoveries which focus on the role of alternative splicing in cardiovascular disease (CVD).

**Figure 2:**
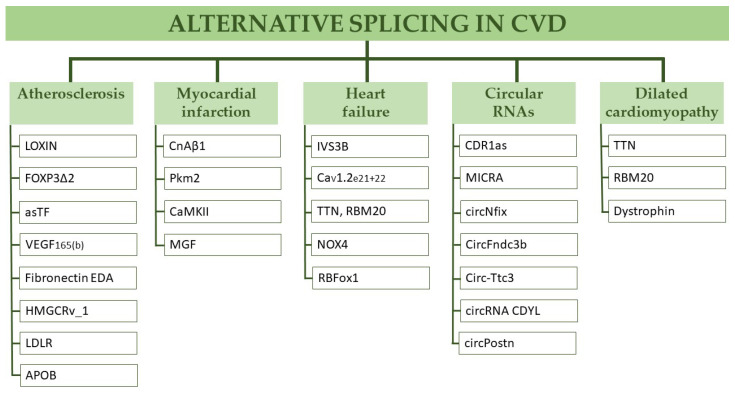
A graphic illustration of the topics mentioned in this review. Abbreviations: CVD—cardiovascular disease. The individual abbreviations are defined upon the first mention of the expression in the main text.

An overview of the topics mentioned in this review is shown in Figure 2.

## 2. Alternative Splicing in Cardiovascular Disease

### 2.1. Atherosclerosis

The term atherosclerosis refers to the gradual thickening of the arterial vessel wall that can eventually result in coronary artery disease, stroke, or myocardial infarction (AMI), and whose progression is affected by a range of well-established lifestyle factors [11,12]. The underlying pathophysiological processes include an endothelial cell inflammatory stimulus, the recruitment of inflammatory cells and the consumption of lipid molecules by macrophages, followed by the formation of foam cells and macrophage death. The final stages involve a further increase in the inflammatory response stimulated by matrix metalloproteinases which affect the stability of the plaque’s fibrous cap, which can finally result in its rupture and an ensuing thrombotic closure of the affected vessel [12].

The link between low-density lipoprotein (LDL) cholesterol and atherosclerosis is a well-established fact, and targeting these particles is one of the pillars in the prevention of adverse cardiovascular events [13,14]. A 2008 study by Medina et al., examined the role of an alternatively spliced variant of the rate-limiting enzyme in the synthesis of cholesterol—HMG-CoA-Reductase (HMGCR) in the level of response to therapy with statins. The alternatively spliced variant at the center of their study was HMGCRv_1, formed by exon exclusion of the exon 13. For their experiments, they used lymphocytes obtained from patients who had previously been subjected to treatment with statins as part of another study to link their in vitro findings to the effectiveness of statin therapy in human patients. They found that a greater induction of HMGCRv_1 following in vitro treatment with statins was linked to a poor lipid response to statin therapy in the patients, whereas no such link could be established for the full-length transcript. They were also able to demonstrate, via a small interfering RNA (siRNA)-mediated knockdown of the full-length variant in vitro, that the activity of the alternatively spliced enzyme was less responsive to statin therapy [15]. In a study conducted in 2014, Yu et al. described the splicing factor heterogeneous nuclear ribonucleoprotein A1 (HNRNPA1) as a key regulator in the alternative splicing of exon 13 in HMGCR, increasing the level of the alternatively spliced transcript without exon 13 in relation to the amount of the full-length transcript. Furthermore, an overexpression of HNRNPA1 depleted the activity of HMGCR [16].

A 2013 study by Gao et al. explored the impact of rs688, a single nucleotide polymorphism of exon 12 of the gene encoding the low-density lipoprotein receptor (LDLR) on its function. Their findings from in vitro experiments on lymphocytes from statin-treated patients confirmed that rs688 regulates the alternative splicing of exon 12 in LDLR and indicated that the alternatively spliced variant is subject to rapid degradation [17]. A previous study by Zhu et al. had linked rs688 to increased LDL-cholesterol levels, with a particularly pronounced increase in premenopausal women [18].

A 2011 study by Medina et al. concluded that the alternative splicing of both previously discussed targets: LDLR and HMGCR, as well as the alternative splicing of further molecules with roles in lipid handling, is mediated in a sterol-dependent manner, adding another layer of complexity to the role of alternative splicing in lipid handling and atherosclerosis [19].

The search for newer therapeutic targets for the prevention of atherosclerosis and lowering of lipid levels has led to new potential targets, some of which aim to harness the power of alternative splicing. In 2007, Khoo et al. conducted a study with a focus on Apolipoprotein B (APOB), a key component of lipid-containing atherogenic particles. They used antisense nucleotides to target splice sites on exon 27 of the gene encoding APOB in order to induce alternative splicing in the form of exon skipping of exon 27. They were indeed able to induce exon 27 skipping, which resulted in the formation of a polypeptide which was not degraded through nonsense-mediated decay. They proposed that this could, in the long run, be a potential approach to lowering blood lipids, through reducing the levels of the full-length isoform [20]. In 2013, Disterer et al. adapted the previously described splice-switching nucleotides targeting exon 27 in order to increase their efficiency and then administered them to transgenic mice who express human Apolipoprotein B. Apart from validating the skipping efficiency in the in vivo model, they also successfully demonstrated a drop in plasma LDL in vivo. Their findings represent a potential avenue of treatment for patients with familial hypercholesterinemia, but also illustrate the feasibility of a targeted induction of exon skipping in vivo [21].

The role of LOXIN, a splicing variant of the Oxidized Low-Density Lipoprotein Receptor 1 (OLR1) gene, generated by exon skipping of exon 5, in atherosclerosis was first examined in 2005 by Mango et al. [22]. The lectin-like oxidized low-density lipoprotein receptor-1 (LOX-1) mediates the uptake of oxidized LDL (oxLDL) particles and their accumulation in the wall of arterial vessels and was found to be enriched in atherosclerotic plaques, as well as play a role in multiple stages of plaque formation and progression, from the induction of a proinflammatory state and the formation of foam cells, to the proliferation of vascular smooth muscles cells [23]. Mango et al. demonstrated that single nucleotide polymorphisms in the non-coding region of the OLR1 gene affect the relative abundance of LOX-1 and LOXIN, its alternatively spliced variant, in macrophages. These SNPs had previously been found to be associated with an increased risk of myocardial infarction in humans [22]. Additionally, Mango et al. found that an increased LOXIN expression in macrophages results in decreased levels of apoptosis and postulated that this might result in increased plaque stability and a lower likelihood of plaque rupture [22]. Three years later, Biocca et al. described a deficiency in the binding of oxLDLby the alternatively spliced LOXIN, as well as an impaired transmembrane localization [24]. They also proposed that LOXIN exerts its beneficial effects through a hetero-oligomerization of LOXIN with LOX-1, with a resulting formation of a non-functional hetero-oligomer [24]. In 2011, White et al. used an apolipoprotein E (ApoE) knockout murine model to demonstrate that an adenoviral gene transfer of LOXIN alleviates the pro-atherogenic effects induced by a LOX-1 gene transfer through the same route [25]. Veas et al. induced apoptosis in human endothelial progenitor cells by treating the cells with oxLDL, which resulted in an upregulation of LOX-1 and dose-dependent apoptosis with higher oxLDL concentrations [26]. The increased level of apoptosis could successfully be prevented through an adenoviral LOXIN gene transfer. [26] Thus, LOXIN, an alternatively spliced variant of LOX-1, appears to convey antagonistic effects, and thus seemingly has beneficial effects. The association of single nucleotide polymorphisms of ORL1 with adverse cardiac events in humans, taken together with the promising findings from animal studies make LOXIN a promising target for treating atherosclerosis.

The observation that Forkhead box protein P3 (FOXP3) is present in multiple isoforms in humans but not in murine models, lead Joly et al. to examine the functional role of alternative splicing isoforms of FOXP3 in humans in the setting of atherosclerosis [27]. A subset of regulatory T cells constitutively express FOXP3 and are essential for developing a self-tolerance in the normal human immune system, and their functional impairment can result in a range of conditions characterized by a maladjusted immune response [28]. Depleted numbers of FOXP3+ regulatory T cells have been found in vulnerable atherosclerotic plaques and in patients who experienced acute coronary syndrome [29]. With regards to the stability of atherosclerotic plaques, regulatory T cells seem to convey a range of non-lipid-related beneficial effects including a decreased immune cell recruitment to the lesion site [29]. In the study by Joly et al., a particular focus was placed on the FOXP3 isoform FOXP3Δ2, which lacks exon 2 and is, together with the full-length transcript, the most abundant isoform in humans. They found an increased expression of FOXP3Δ2 in activated regulatory T cells and a decreased expression of FOXP3Δ2 was linked to plaque instability in patients. Furthermore, their findings indicated that the full-length transcript and the isoform lacking exon 2 did not have a fullfunctional overlap. [27] Based on these findings, the authors proposed a potential complementary role of FOXP3 alternative splicing as an add-on to other regulatory T-cell based therapeutic approaches [27]. An increased relative abundance of the splicing isoform FOXP3Δ2 was also found in patients with coronary artery disease in another study [30].

The role of the alternatively spliced tissue factor (asTF) in atherosclerosis was examined by Giannarelli et al. [31]. Aside from its well-known role as the starting step of the extrinsic coagulation pathway, tissue factor (TF) has also been found to be highly present in atherosclerotic plaques, although its role in the formation or progression of the plaques is not fully clear [32]. Giannareali et al. initially demonstrated that asTF is highly expressed in complicated atherosclerotic human lesions, as compared to uncomplicated ones. Their ensuing in vitro experiments revealed that it also induces the powerful angiogenetic hypoxia-induced factor 1-α (HIF-1α). Finally, they discovered an increase in the expression of the vascular endothelial growth factor isoform 165 (VEGF165) in cells where HIF-1α was stimulated through asTF. In a murine model, animals with an induced asTF overexpression were more prone to an increased neovessel formation in the plaques. Taken together, these results imply that the alternatively spliced tissue factor might play a role in the formation of complicated plaques, possibly through a locally pronounced increase in angiogenesis, and that some of its effects appear to be conveyed through HIF-1α [31]. In a different study, asTF was also found to be more potent in inducing cellular adhesion molecules and thus promoting the adhesion and migration of monocytes across the endothelial layer than its full-length counterpart [33].

Even for factors with a previously established role in atherosclerosis, examining alternative splicing products can offer additional insights. Zhao et. al. demonstrated that, in their ApoE knockout murine model of atherosclerosis, the proangiogenic VEGF165 was the preferred VEGF-A splicing variant, both in aortic endothelial cells and in macrophages [34]. Further in vitro experiments led them to the conclusion that this shift in splicing is most likely the result of an inhibition of anti-angiogenic splicing via the serine/arginine-protein kinase (SRPK1). The proangiogenic splicing shift induced increased apoptosis of macrophages and proliferation of endothelial cells [34]. Thus, they concluded that alternative splicing pathways, and particularly the splicing variant VEGF165 may play a role in the formation of atherosclerotic plaques. Its antiangiogenic counterpart, VEGF165b, which is formed through alternative splice site selection on exon 8, induces an antiangiogenic phenotype in macrophages, which, as shown by Ganta et al., impairs the recovery of perfusion of ischemic muscle in an animal model of limb ischemia [35].

Interestingly, it is not always one specific alternative splicing variant that plays an important role in the pathogenesis of a disease, but sometimes, as shown in the example of fibronectin in a study by Babaev et al. the act of alternative splicing itself, with two or more resulting products whose combined effect differs from both individual ones. Babaev et al. set out to examine the role of an alternatively spliced fibronectin variant FibronectinEDA, which contains the extra domain A (EDA), and is a significant component of the extracellular matrix [36]. Fibronectin is present in plasma and cellular forms and conveys cell and molecular adhesion and different extracellular matrix properties, and its numerous alternatively spliced variants have been identified over two decades ago [37]. FibronectinEDA had been found to be enriched in atherosclerotic plaques, which lead Babaev et al. to create two strands of mice that were unable to perform EDA exon regulated splicing and either constitutively included or excluded the EDA exon [36]. They were surprised to find that both mice strands, rather than only the one which constitutively excluded the EDA exon, were less susceptible to the formation of atherosclerotic plaques compared to the control animals [36]. This led them to conclude that it was the controlled process of splicing, and not EDA on its own, that play a role in atherosclerosis. However, in contrast with these findings, constitutively EDA deficient mice were found by Cappellari et al. to suffer from a greater degree of endothelial dysfunction and oxidative stress compared to the constitutively EDA expressing and control animals in a murine streptozotocin-induced diabetes model [38]. Although these findings are not in direct conflict with the initially reported results due to the use of a different model whose primary use is studying diabetes-related endothelial dysfunction, they warrant further research on the role of FibronectinEDA in atherosclerosis.

### 2.2. Myocardial Infarction

Acute myocardial infarction (AMI) is the result of a ruptured atherosclerotic plaque which results in the spillage of the thrombogenic contents into the lumen that drive the formation of a thrombus which subsequently becomes trapped in the narrower distal part of the vessel and causes ischemia for the section of the myocardium that is supplied by the occluded branch [39]. Although rapid revascularization strategies have greatly improved post-AMI outcomes, AMI often leads to an adverse remodeling of the left ventricular myocardium which might, in the long run, ultimately lead to heart failure [40].

Calcineurin Aβ1 (CnAβ1) is an alternatively spliced variant of Calcineurin Aβ, one of the three naturally occurring isoforms of calcineurin. Felkin et al. set out to explore its role in the heart after they encountered its regeneration inducing and inflammation resolving function in skeletal muscle [41,42]. CnAβ1 is an alternatively spliced product that lacks the autoinhibitory domain present in the CnAβ2 variant, which binds to the active site of the catalytic subunit of calcineurin under steady-state conditions and only detaches upon a significant increase in intracellular calcium levels. CnAβ1 was induced postnatally in a transgenic murine model where its expression stood under the control of an αMHC promoter. Thus, its overexpression could be induced in the cardiomyocytes postnatally. The mice, together with the wild-type control mice, then underwent an experimental MI through the ligation of the left anterior descending artery (LAD), as well as a series of follow-up examinations. The area of the resulting scar, the deposition of collagen, the hypertrophy of the cardiomyocytes, the contractile and diastolic dysfunction were all lower in the mice with an induced overexpression of CnAβ1. Their results also indicated that CnAbeta1 activates Transcription Factor 4 (TF4) and a range of cardioprotective pathways, as well as the Akt pathway [41]. The main potential advantage of CnAβ1 as a potential therapeutic target arising from this study is that it might shield the myocardium from ischemia related injury without resulting in a pathologic degree of hypertrophy. The range of beneficial effects conveyed by CnAβ1 was also investigated in a newer study by Padrón-Barthe et al. in a murine model of pressure overload hypertrophy. Aside from determining that CnAβ1 has a protective effect against cardiac hypertrophy in this setting, they also showed that CnAβ1 activates one-carbon metabolism, resulting in an increased supply of ATP and glutathione to the myocardium, resulting in an improved function and greater resistance to reactive oxygen species [43].

Williams et al. also used next-generation sequencing to assess the alternative splicing changes in response to a surgically induced murine MI, and focused on pyruvate kinase, a key glycolysis enzyme. They detected an upregulation of pyruvate kinase isoform Pkm2, which, in contrast to Pkm1, includes exon 10 rather than exon 9. The upregulation of Pkm2 in relation to Pkm1 persisted during the remodeling stage as well, and the authors postulated that this represents an adaptive metabolic response to hypoxia, as the Pkm2 isoform disfavors oxidative phosphorylation. Their findings also indicated a role for HIF-1 in mediating the post-infarction shift between the two isoforms [44].

In a recent publication, Williams et al. focused on a different aspect of the HIF-1α mediated alternative splicing under ischemic conditions. They compared the alternative splicing modifications in a line of mice with an induced expression of an oxygen-stable HIF-1α to control mice that had undergone an experimental MI and placed a focus on calcium handling genes. They indeed detected alternative splicing patterns of the calcium/calmodulin-dependent protein kinase II (CaMKII) in both cases, with a decrease in the long v1 variant and the related splicing factor Rbfox1. According to the authors, this implies an impact of HIF-1α on calcium handling in hypoxic or ischemic conditions through alternative splicing [45].

Mavrommatis et al. explored the role of the E-domain region of the mechano-growth factor (IGF-1Eb), an isoform of the insulin-like growth factor 1 (IGF-1) formed by alternative splicing, in the response to MI. The expression of this isoform was indeed increased in response to cell stress in cell culture and a peptide treatment with the E-domain reduced apoptosis in this setting. The delivery of the stabilized peptide to mice following MI attenuated the functional impairment of the murine heart [46]. A previous study had also detected a comparatively higher increase in the mechano-growth factor following myocardial infarction in mice compared to the increase in IGF-1Ea [47].

### 2.3. Heart Failure

Heart failure represents the final chronic stage of a multitude of cardiac diseases which is characterized by an impairment of the effective filling of ventricles with blood or a reduced ability to pump the blood out of the ventricle [48]. Heart failure is most commonly classified according to the ejection fraction, which illustrates whether a contractile or a filling impairment is the primary cause of the observed clinical symptoms [48].

A study by Yang et al. examined the abundance of the isoforms of the third transmembrane spanning the region of the fourth domain of the α1c gene which encodes the α1 subunit of the L-type voltage-activated calcium channels in the heart. These channels are, among other things, crucial for proper excitation–contraction coupling and have been found to play a role in hypertrophy and heart failure [49]. The isoforms are the results of alternative splicing and contain either exon 31 or 32. Yang et al. found that their abundance varies between cardiomyocytes collected from patients with failing hearts at the time of heart transplantation and samples from non-failing hearts [49]. A 2016 study by Hu et al. examined the significance of another alternative splicing event that also affects cardiac L-type calcium channels [50]. In their rat model, they discovered a variant of the L-type voltage-gated calcium channel formed by alternative splicing via the inclusion of exons 21 and 22 (Cav1.2e21 + 22), whose expression varies between neonatal and adult hearts, with a higher expression in the neonatal state, and whose presence was also increased in a rat model of cardiac hypertrophy and heart failure [50]. They found that this variant affects the presence of the wild-type calcium channels, and results in their degradation through a competitive interaction with the β subunit of the L-type voltage-gated calcium channels [50]. In 2017, Li et al. examined the significance of an alternatively spliced variant of the L-Type Calcium voltage-dependent channel which lacks exon 33 and had previously been found to be upregulated in rat hearts following MI [51]. The exon 33 knockout mice were found to be prone to ventricular tachycardia, arrhythmia and to death following β adrenergic stimulation [51]. A follow-up study found that a heterozygous knockout of exon 33 results in cardiomyocytes whose calcium channel properties are similar to those of WT mice and that these cardiomyocytes exhibit normal membrane excitation patterns [52]

Titin, whose role in DCM we discuss in the following segment, has also emerged as a novel target for improving diastolic dysfunction [53,54]. Motivated by a 2015 study by Zile et al., that found changes in the titin-dependent stiffness of the myocardium in patients suffering from heart failure with a preserved ejection fraction (HFpEF), Bull et al. designed a study that examined the potential benefit of altering the prevalence of titin isoforms on improving diastolic dysfunction. This was achieved by depleting the titin splicing factor RBM20 [53,54]. Inhibiting RBM20 in a mouse model of diastolic dysfunction resulted in an improvement of the diastolic function that seemed to be achieved by increasing the number of compliant titin isoforms generated by alternative splicing in the spring region of titin [54].

Varga et al. studied the alternative splicing of the NADPH oxidase 4 (NOX4) and its regulation in failing human hearts. NOX4 is an enzyme known to produce reactive oxygen species (ROS), which have been implicated in the progression of heart failure [55]. They detected multiple splicing variants in failing human hearts, with a higher prevalence of the full-length isoform in patients whose heart failure developed on the basis of an ischemic cardiomyopathy [55].

Gao et al. detected a reduced expression of the RNA binding protein FOX-1 (RBFox1) in a murine model of pressure-induced heart failure [56]. Furthermore, they detected numerous RNA splicing changes in murine cardiomyocytes with an adenovirally induced expression of RBFox1. They found that the induced expression of RBFox1 affects the inclusion of mutually exclusive exons in the transcription factor MEF2 [56]. Finally, they found that RBFox1 knockout mice were more prone to developing heart failure and the accompanying structural changes. Stimulating RBfox1 in mice with an inducible expression of RBFox1 was protective against cardiac hypertrophy and the loss of contractile function in response to pressure overload [56].

### 2.4. Dilatative Cardiomyopathy

Dilated cardiomyopathy (DCM) belongs to the category of structural heart diseases and is non-valvular, non-hypertensive and non-ischemic in origin [57]. It is characterized by a dilation of the ventricles and a concomitantly reduced systolic function and is a frequent cause of heart failure [57]. Approximately one-third of all DCM cases appear to have an underlying primary genetic cause [58].

In a landmark study published in 2012, Herman et al. used next-generation sequencing to uncover a link between the gene encoding titin (TTN), the largest known protein responsible for passive muscle stiffness, and DCM [59,60]. However, the described changes that were found with a greater frequency in DCM patients, were the result of conventional truncating mutations, rather than alternative splicing events. That same year, Guo et al. discovered that the RNA binding motif 20 (RBM20) was responsible for regulating the alternative splicing of the PEVK region of TTN. They identified the loss of the RBM20 as the cause of a TTN splicing deficiency in a mice strain known to exhibit a DCM-like phenotype, which was characterized by unusually large titin molecules. They found that RBM20 was responsible for the regulation of titin isoform expression and discovered larger diastolic left ventricle diameters in rats with a heterozygous or homozygous loss of the RBM20 allele [61]. Finally, they identified over 30 genes whose alternative splicing was regulated in an RBM20-dependent manner [61]. Even prior to this publication, RBM20 mutations had been found to be associated with DCM, but the downstream events as a consequence of such mutations had not been examined in great detail [62]. In 2016, Khan et al. described numerous circular RNAs (circRNAs), a product of backsplicing, that arise from the TTN gene using whole transcriptome sequencing in six human patients [63]. Using RBM20 knockout mice that developed DCM, they were able to prove that two subsets of circRNAs are formed from TTN—a subgroup whose formation was independent of RBM20 and a subset located in the I-band region whose formation required the activity of RBM20, thus indicating that RBM20 is only essential for the production of circRNAs from a certain region of the TTN gene [63]. In 2017, Bömeke et al. used skin punch biopsies from a patient with severe DCM who had a missense S635A mutation in RBM20 to obtain fibroblasts, to then generate induced pluripotent stem cells and induce their differentiation into cardiomyocytes. These cardiomyocytes showed an altered distribution of sarcomeric actin following differentiation and were used to engineer heart muscle and compare its properties to those of the DCM patient. By doing this, they discovered a comparable impairment in active contractile force and a decreased stress-strain response in the engineered muscle. Finally, the patient-derived induced pluripotent stem cells demonstrated a deficient alternative TTN splicing and switching of TTN isoforms. [64] Recently conducted research by Briganti et al. discovered a potential therapeutic principle for patients suffering from severe DCM forms that are a consequence of RBM20 mutations. Treatment with all-trans retinoic acid (ATRA) was used in vitro on induced pluripotent stem cell-derived cardiomyocytes derived from a patient with DCM and a known RBM20 mutation and managed, in this setting, to improve the splicing defects and the impaired contractility. The beneficial effects required a residual RBM20 activity, but as human patients always have one functional allele, this does not limit the applicability of the therapy [65]. This example illustrates the numerous levels of regulation exerted via alternative splicing, even on the example of a comparatively rare condition such as DCM. Furthermore, the in vitro results from the study by Briganti et al. represent a potential target for treating a condition with an otherwise perilous natural course.

A particular subset of DCM is encountered in a group of muscular disorders encompassed by the term dystrophinopathies, which are caused by mutations in the DMD gene which lead to the lack of a functional form of the dystrophin protein. Dystrophin is part of an intracellular complex that connects the extracellular matrix to the actin cytoskeleton and stabilizes the plasma membrane during contractile activity, and the lack of this critical component results in an increased vulnerability of the plasma membrane. In terms of cardiac involvement, this results in the phenotype of a dilated cardiomyopathy with progressive fibrosis [66]. Until this day, no widely available treatments have been established and available pharmacological therapies did little to curb the progression of the disease. However, a landmark study conducted in 2004 by Lu et al. used antisense oligonucleotide-mediated alternative splicing induced via the systemic administration of 2′-O-methyl phosphorothioate antisense oligoribonucleotides (2OMeAOs) in a murine model of muscular dystrophy, which is characterized by a nonsense point mutation of exon 23, to successfully induce targeted alternative splicing, which resulted in an expression of dystrophin in all skeletal muscles [67,68]. Three years later, in a small-scale human trial by Deutekom et al., patients received antisense oligoribonucleotides targeting exon 51, and a successful induction of dystrophin expression through exon skipping was verified in biopsy samples from the participants [69]. These findings led to the first pharmacological trials in humans, such as the one conducted with Drisapersen, an exon-51-targeting antisense oligonucleotide, which resulted in the improvement of several functional parameters of muscular dystrophy patients in a Phase II trial, although the Phase III trial findings were not significant, possibly due to inclusion strategies and the study design [70,71]. However, long-term large-scale study data are still necessary to deliver the verdict on this promising avenue of treatment.

### 2.5. Circular RNAs in Myocardial Infarction

Circular RNAs (circRNAs), are covalently closed-loop RNA molecules formed through the ligation of a downstream donor site with an upstream splice acceptor site. [72] Their early discovery was followed by a stagnant phase where it was largely assumed that they had little functional significance, which lead to them being left largely unexplored in the ensuing decades [72]. Advances in high-throughput sequencing revealed their relative abundance and conservation across species, which lead to a renewed interest in the field [72]. Some of the discoveries pertaining to the role of circRNAs in the setting of myocardial infarction are detailed in this section. An overview of the mentioned circRNAs, the models used to examine them in the studies listed below and the proposed targets can be found in Table 1.

In a landmark paper published in 2016, Geng et al. detected an increased expression of the circular RNA CDR1as in a murine MI model, but also in cardiomyocytes in cell culture following a period of hypoxia [73]. Their research was motivated by a previous study conducted by this group of researchers that found that miR-7a and miR-7b was protective in the setting of ischemia-reperfusion injury in cell culture through the inhibition of the proapoptotic polyADP-ribose polymerase (PARP) [74] CDR1as, on the other hand, had been found to act as a miR-7 sponge in a different publication [75]. In their paper, Geng et al. detected an upregulation of miR-7a, that has a regulatory impact on cell apoptosis following ischemia/reperfusion injury. The overexpression of miR-7a seemed to protect the cells from a previously induced CDR1as-mediated apoptosis in cell culture. miR-7a achieved these effects by downregulating PARPand the transcription factor SP1, which had previously been upregulated by CDR1as. The authors thus seemed to confirm that CDR1as acts as a miR-7a sponge. [73] In 2020, Mester-Tonczar et al. successfully detected CDR1as in pig hearts following an experimental MI. However, in this study, and in contrast with the abovementioned findings, CDR1as had a positive impact on the infarct size and the recovery of functional parameters [76]. Thus, these conflicting findings warrant further research on the role of CDR1as in the setting of MI, and in large animal models in particular.

In 2016, Vausort et al. collected peripheral blood samples from patients after reperfusion following an AMI in order to assess the potential of circRNAs for outcome prediction following revascularization. They discovered a myocardial infarction-associated circular RNA (MICRA) which was differentially regulated in patients following the infarction as compared to healthy volunteers. They found that assessing its levels in peripheral blood could help predict long-term left ventricular dysfunction [77]. This example illustrates the potential diagnostic value of circRNAs, whose stability makes them attractive diagnostic research targets, and whose predictive capabilities might possibly surpass those of established laboratory markers.

In 2019, Huang et al. identified circRNA Nfix (circNfix) that is regulated by a superenhancer, a region with a prominent regulatory activity and a strong clustering of areas that bind coactivators of transcription. circNfix was found to be enriched in cardiomyocytes. An adeno-associated virus-9 short hairpin RNA (shRNA)-mediated knockdown of circNfix in mice resulted in an increased proliferation of cardiomyocytes. A local knockdown of circNfix induced by an injection of a shRNA into the periinfarction area of adult mice promoted cardiac regeneration and attenuated myocardial dysfunction. circNfix was found to act as a sponge for miR-214 [78]. These findings make circNfix a promising target for future therapeutic approaches for ischemic heart disease.

Another candidate circRNA with promising in vivo results is CircFndc3b [79]. The expression of circFndc3b, which was found to be decreased not only in the period immediately following myocardial infarction in mice, but also over an extended follow-up period, stood in contrast with the linear transcript, whose expression remained unchanged [79]. After observing the beneficial effects of CircFndc3b on cell survival in cell culture, an Adeno-associated virus-9-mediated cardiac overexpression of circFndc3b administered through an intramyocardial injection resulted in a less pronounced left ventricular dysfunction and less scar tissue formation in mice following an experimental myocardial infarction [79]. CircFndc3b appears to act by regulating VEGF signaling [79].

In 2019, Cai et al. examined the role of Circ-Ttc3, one of the most highly expressed cardiac circRNAs, in in vitro and in vivo models of hypoxia and ischemia [80]. After demonstrating that hypoxia induced the upregulation of Circ-Ttc3 and after in vitro experiments had successfully demonstrated that Circ-Ttc3 is protective with regards to hypoxia-related apoptosis, the group then conducted experiments using a recombinant adenoviral-associated vector-cTnt-mediated downregulation of Circ-Ttc3 [80]. The downregulation of Circ-Ttc3 exacerbated post-infarction remodeling in mice, suggesting a protective effect of Circ-Ttc3 on ischemia-related injury [80]. Finally, Cai et al. proved that Circ-Ttc3 acts as a sponge for miR-15b, and that the previously observed protective effects seem to be conveyed through this axis [80]. In the murine model of another study, the inhibition of miR-15b following ischemia reperfusion injury resulted in a greater functional improvement following the event, and reduced the level of cardiac remodeling [81].

The role of circRNA CDYL in myocardial infarction was examined by Zhang et al. in 2020. In vitro experiments revealed that circRNA CDYL can promote and inhibit the proliferation of cardiomyocytes. Again, an upregulation of circRNA CDYL was induced via the intramyocardial application route following MI in mice and resulted in an improvement of the functional parameters. The regulation of cell proliferation exerted by circRNA CDYL were found to be achieved by sponging miR-4793-5p [82].

CircRNA circPostn, on the other hand, seems to aggravate myocardial injury following myocardial infarction, as described by Cheng et al. in 2021 [83]. They detected increased circPostn levels in plasma samples from human individuals following AMI and in myocardial tissue samples from post-infarction mice. Afterwards, they used a lentiviral circPostn shRNA vector to deplete circPostn in mice on the third day after MI [83]. This approach resulted in less pronounced cardiac remodeling [83]. circPostn was found to act as a sponge for miR-96-5p [83]. The precise role of this miRNA in the setting of cardiovascular disease is still largely unexplored.

The numerous discoveries obtained over a comparatively short timeframe illustrate the untapped potential of circRNAs in cardiovascular research.

## 3. Conclusions

Although the concept of alternative splicing has been around for many decades, it is only in recent years, with the advent of high throughput approaches and novel analytic methods, that we are beginning to grasp the extent to which it affects and further complexifies the pathophysiology of various conditions, including cardiovascular disease.

In this review, we attempted to provide an insight into some of the many ways alternative splicing is involved in atherosclerosis, heart failure, myocardial infarction, and dilated cardiomyopathy. circRNAs are a relatively new yet highly promising avenue of research on cardiovascular diseases, even though we are surely only beginning to scratch the surface of the number and functions of circRNAs in cardiovascular disease. Whereas some pathways involving alternative splicing are only just being elucidated, other alternative splicing targets have been successfully examined in small and large animal studies, and some, perhaps best illustrated by the therapeutic approaches targeting dystrophin, are well on their way to potentially becoming available for use in patients in the foreseeable future.

## Figures and Tables

**Table 1:** circRNAs mentioned in this review. Abbreviations: circRNA; MICRA—myocardial infarction-associated circular RNA; VEGF—vascular endothelial growth factor.

circRNA	Model	Target
CDR1as	Mouse, pig	miR-7a
MICRA	Human	miR-150, not definitively proven
circNfix	Mouse	miR-214
CircFndc3b	Mouse	VEGF signaling
Circ-Ttc3	Mouse	miR-15b
circRNA CDYL	Mouse	miR-4793-5p
circPostn	Mouse	miR-96-5p
